# Influence of Light Conditions on Microalgae Growth and Content of Lipids, Carotenoids, and Fatty Acid Composition

**DOI:** 10.3390/biology10101060

**Published:** 2021-10-18

**Authors:** Yevhen Maltsev, Kateryna Maltseva, Maxim Kulikovskiy, Svetlana Maltseva

**Affiliations:** 1 Laboratory of Molecular Systematics of Aquatic Plants, K.A. Timiryazev Institute of Plant Physiology RAS, IPP RAS, 127276 Moscow, Russia; max-kulikovsky@yandex.ru (M.K.); svetadm32@gmail.com (S.M.); 2 Faculty of Chemistry and Biology, Bogdan Khmelnitsky Melitopol State Pedagogical University, 72312 Melitopol, Ukraine; katti_m@ukr.net

**Keywords:** carotenoids, fatty acids, light, lipids, microalgae, photoperiod

## Abstract

**Simple Summary:**

Microalgae are a valuable natural resource for a variety of value-added products. This determines the relevance of developments aimed at increasing the efficiency of their cultivation. First of all, the growth of microalgae is determined by the impact of light. The main parameters characterizing the light conditions during the cultivation of microalgae are light intensity, duration of lighting, and the use of rays of different spectral composition. Manipulations with the duration of the photoperiod and the use of pulsed light can avoid photodamage of microalgae cells and create conditions for the most efficient absorption of light photons. The optimal light conditions for photosynthesis, growth, accumulation of lipids, fatty acid composition, and carotenes content differ both in different taxonomic groups of microalgae and in different strains of the same species.

**Abstract:**

Microalgae are a valuable natural resource for a variety of value-added products. The growth of microalgae is determined by the impact of many factors, but, from the point of view of the implementation of autotrophic growth, light is of primary importance. This work presents an overview of the influence of light conditions on the growth of microalgae, the content of lipids, carotenoids, and the composition of fatty acids in their biomass, taking into account parameters such as the intensity, duration of lighting, and use of rays of different spectral composition. The optimal light intensity for the growth of microalgae lies in the following range: 26−400 µmol photons m^−2^ s^−1^. An increase in light intensity leads to an activation of lipid synthesis. For maximum lipid productivity, various microalgae species and strains need lighting of different intensities: from 60 to 700 µmol photons m^−2^ s^−1^. Strong light preferentially increases the triacylglyceride content. The intensity of lighting has a regulating effect on the synthesis of fatty acids, carotenoids, including β-carotene, lutein and astaxanthin. In intense lighting conditions, saturated fatty acids usually accumulate, as well as monounsaturated ones, and the number of polyunsaturated fatty acids decreases. Red as well as blue LED lighting improves the biomass productivity of microalgae of various taxonomic groups. Changing the duration of the photoperiod, the use of pulsed light can stimulate microalgae growth, the production of lipids, and carotenoids. The simultaneous use of light and other stresses contributes to a stronger effect on the productivity of algae.

## 1. Introduction

Microalgae (eukaryotic and prokaryotic cyanobacteria) are a large group of photoautotrophic organisms, as well as organisms capable of heterotrophic, mixotrophic, and photoheterotrophic existence [1,2]. They are adapted to life in a variety of habitats and produce biomass containing a wide variety of compounds that can be used in the food, pharmaceutical, and cosmetic industries, and for the production of biofuels, etc. [3,4,5,6]. Of significant interest to the food and feed industry is the ability of microalgae to produce carotenoids such as β-carotene, astaxanthin, lutein, as well as lipids containing a wide variety of fatty acids, including long-chain polyunsaturated fatty acids (PUFAs) from the omega-3 and omega-6 [7,8,9,10,11,12,13] group.

To meet the needs of the market for these products, an extremely important task is to find and introduce highly productive microalgae species into the culture, to determine the optimal cultivation conditions and technologies that meet the requirements of commercial feasibility. Recent reviews reveal major advances in research on the ability of microalgae to produce carotenoids, lipids, valuable fatty acids, and the possibilities and prospects of metabolic engineering to increase their production [13,14].

It has also been reported about the great possibilities of regulating the yield of commercially valuable bioproducts when using the main abiotic stress factors during the cultivation of microalgae [15,16,17,18]. The main achievements in this regard have been generalized in a number of recent reviews [11,19,20,21]. These reviews discuss the main results of the influence of all the main abiotic factors: changes in lighting and cultivation temperature, limitation of nitrogen and phosphorus in nutrient media, the use of salt stress, ultraviolet and magnetic radiation, the influence of heavy metals on the growth rate of microalgae, the productivity of lipids, carotenoids, fatty acids. The concentration in one work of all the variety of stress factors is very valuable in terms of a systemic approach to this problem, but inevitably leads to certain limitations associated with the inability to enter into details.

The growth of microalgae is determined by the action of many factors, but, from the point of view of the implementation of autotrophic growth, light is of primary importance. It determines the functional state, growth, and reproduction of photoautotrophic microalgae and plants, and also has a direct effect on the metabolism of both mixotrophic microalgae, capable of combining autotrophic and heterotrophic types of nutrition, and photoheterotrophic ones [22,23,24].

In natural habitats, the light conditions for the growth of microalgae are determined by the geographical latitude of the area, daily and seasonal changes in the solstice altitude, light scattering by the atmosphere, water column, etc. and are very variable. Accordingly, microalgae are adapted to various lighting conditions. There are species that are found in highly shaded habitats, for example, in the soil under the dense canopy of higher plants [25,26], above-water and underwater caves, in deep water layers [27,28,29]. Other species are adapted to growth in direct sunlight, for example, in polar, tropical deserts [30,31,32], on salt marshes and other open surfaces [33,34,35,36], where the light intensity can reach 2000 µmol photons m^−2^ s^−1^ [15].

When cultivating microalgae, both natural and artificial lighting are used. Natural light is used for growing microalgae in open systems and cannot be purposefully controlled. In closed growing systems—photobioreactors—artificial lighting is applicable. In this case, the lighting conditions can be changed and this can be used to increase the productivity of microalgae biomass and the accumulation of valuable compounds in it. An urgent task in the microalgae cultivation is to minimize the energy costs for providing lighting, since they largely determine the cost of compounds obtained from microalgae [37]. The main parameters characterizing the lighting conditions are light intensity, duration of lighting, and the use of rays of different spectral composition. These parameters can be changed individually and in various combinations when cultivating microalgae.

The purpose of this article is to summarize information on the effect of lighting conditions on the growth of microalgae, the content of lipids, carotenoids, and the composition of fatty acids in their biomass, taking into account such parameters as the intensity, duration of lighting and the use of rays of different spectral composition. We assume that the information provided will be valuable for further developments to increase the efficiency of cultivation of microalgae species and obtain value-added products from their biomass, such as carotenoids, lipids, and fatty acids by changing certain lighting parameters.

## 2. Light as an Environmental Factor of Microalgae Growth

It is known that low lighting can have a limiting effect on the microalgae growth, and too high lighting can have an inhibitory one [38,39,40]. The light intensity directly affects the photosynthesis rate, and this dependence is known to be expressed by a logarithmic curve [41]. At low light intensity, an increase in lighting leads to an almost linear increase in the photosynthesis rate. However, in the region of saturating light intensities, a further increase in lighting does not increase the photosynthesis rate. Microalgae vegetation under excessive light can lead to photo-oxidative damage to the photosynthetic apparatus, and decrease in the efficiency and rate of photosynthesis, i.e. to photoinhibition [15].

The molecular mechanisms of photoinhibition are quite complex. Enhanced production of reactive oxygen species (ROS) can occur during photoinhibition in cells. ROS are highly reactive [21,42]. In small amounts, they perform a signaling function and trigger various mechanisms to counteract stress, but their high concentrations lead to damage of cell structures, transformation of physiological and biochemical processes. In response to oxidative stress, synthesis of antioxidant enzymes, phytohormones, and other photoprotective compounds can be observed in algal cells, due to the oxidation of which, reactions that are more dangerous for the cell can be interrupted [15,42]. Also, as a response to stress there are changes in photosystems, the composition and ratio of the components of the light-harvesting complex, the accumulation of lipids, carotenoids, and the composition of fatty acids [14,43,44,45]. Thus, oxidative stress can significantly improve the commercial characteristics of algal species. In this case, the main task is to achieve an optimal balance between the growth rate of microalgae and induction of compounds valuable from a biochemical point of view.

When discussing the regulatory effect of light on the growth of microalgae, as a rule, not the entire light flux is studied, but that part of it that has the greatest physiological activity. This radiation corresponds to the visible part of the spectrum in the wavelength range 400–700 nm and is called photosynthetically active radiation [46]. When characterizing the light conditions for microalgae growth, the light intensity, duration of lighting and the spectral composition of the light flux are taken into account.

Intensity is a quantitative characteristic of light. There is no common approach to measuring light intensity among researchers. The energy quantitative characteristic of light radiation is the flux of radiant energy, which arrives at the surface perpendicular to the rays per unit of time. To determine the intensity of photosynthetically active light flux, an estimate of the number of photons is widely used, expressing them in micro Einsteins (μE m^−2^ s^−1^) or in micromoles (µmol m^−2^ s^−1^) per unit area per second [18,47,48,49]. The light intensity can also be presented in lux (1 lx = 1 lumen per square meter) or W m^−2^ [50,51]. As the analysis of publications [18,47,48,49,50,51] shows, when studying the effect of light on the growth and production characteristics of microalgae, different units of measurement are also used, which creates certain difficulties in comparing the results obtained. Online calculators can be used to convert different units of lighting radiation. An intensity of 250 lx for the incandescent tungsten halogen lamp equivalents to 5 µmol m^−2^ s^−1^ and 1 W m^−2^. But an intensity of 250 lx for the cool white fluorescent lamp corresponds to 3.5 µmol m^−2^ s^−1^ and 0.763 W m^−2^ [52]. 

The duration of lighting is also of great importance, both from the point of view of ensuring maximum microalgae productivity, and for minimizing energy costs during cultivation. The practice of using continuous lighting or alternating light and dark cultivation cycles is known, including pulsed (blinking) lighting, which is characterized by a rapid change in light and dark periods [46,53,54]. The use of light and dark cultivation cycles is associated with the peculiarities of photosynthesis—a complex multistep process in which light-dependent photochemical reactions and dark reactions are distinguished, wherein the dark reaction of photosynthesis is not dependent on the presence of light [14].

When assessing the effect of lighting conditions on the growth and production characteristics of microalgae, many researchers also pay attention to the cell density in the cultures [38]. High cell density prevents light penetration, which in turn leads to a decrease in the intensity of photosynthesis [55]. To improve the lighting conditions, it is recommended to regulate the cell density, provide the stirring of the cultures, and change the area of the illuminated surface and the shape of the bioreactors, etc. This issue is discussed in detail in several research works [43,45,56].

Algae pigments provide the absorption of light energy. When a light quantum is absorbed, the pigment molecules are excited and is converted into a high-energy state. When they return to their initial state, they release energy due to which photochemical transformations occur [57]. The process of absorption of light energy, the structure of light-harvesting complexes of microalgae, and the features of energy transfer to the reaction centers of photosystems I and II are described in detail [14,56,58]. Each microalgae photosystem contains a distinctive set of light-harvesting pigments that provide a unique absorption spectrum (Table 1).

In addition to chlorophyll *a*, other types of chlorophyll are known in microalgae: *b*, *c*, *d*, and *f* in some cyanobacteria [40,60,61,62]. All chlorophylls are magnesium complexes of various tetrapyrroles [57]. Chlorophylls selectively absorb light in the red and blue spectral region [59]. Moreover, the absorption spectrum of each of the chlorophylls has its own characteristics [66]. Algal cells contain either only chlorophyll *a* or chlorophyll *a* as the main one and other chlorophylls as the second additional pigment [57,62,66].

Phycobilins provide additional light absorption in the 490–650 nm range [65]. The phycobilins of cyanobacteria are not identical to those of Rhodophyta and are named, respectively, C-phycocyanin, C-phycoerythrin and R-phycocyanin and R-phycoerythrin. Cryptophyta phycobilins are subdivided into phycoerythrins (Cr-PE) or phycocyanins (Cr-PC), but neither Cryptophyta species contains both [58]. The spectral characteristics of phycobilins have slight differences in different species [65]. 

Another large group of accessory pigments are carotenoids. The absorption spectra of carotenoids are in the range 400–500 nm [67]. Carotenoids are a widespread group of pigments. Currently, as noted by Wan et al. [13], there are over 1100 carotenoids. Depending on the availability of oxygen, carotenoids are divided into carotenes, pigments that do not contain oxygen, and xanthophylls, pigments that contain oxygen [67]. The most common carotenoids in microalgae are β-carotene and xanthophylls—lutein, violaxanthin, zeaxanthin [20,67]. 

Carotenoids associated with the photosynthetic apparatus of microalgae and providing the absorption of light energy are referred to as primary carotenoids [21]. It is reported that their diversity is significantly lesser than that of secondary carotenoids, which are not associated with the provision of photosynthesis and vary significantly in different taxonomic groups of algae [68]. Secondary carotenoids are often produced by cells in response to specific environmental conditions, such as strong light. They accumulate in the peripheral cytoplasm or plastid stroma [20]. Carotenoid’s characteristic of the photosynthetic apparatus of algae, for example, β-carotene, lutein, can also accumulate as secondary carotenoids [21]. Widely known secondary carotenoids are astaxanthin, the end product of the biosynthesis of secondary carotenoids in a number of microalgae, and canthaxanthin [68,69].

From the ecological and physiological points of view, the different composition of microalgae pigments demonstrates differences in the ability of algae to use light of different spectral composition for photosynthesis and to develop niches with different lighting conditions [18,27,30,70]. Therefore, when cultivating microalgae, the spectral composition of light is an important growth parameter [71,72].

To provide lighting, fluorescent lamps or LEDs are used [73]. Spectrally, the light of a luminescent lamp has maxima in the blue-violet part of the spectrum, valuable from the point of view of ensuring photosynthesis. The red part of the spectrum contains an insignificant part of light energy [74]. Unlike fluorescent lamps, the use of LEDs allows you to create lighting with a certain wavelength, create monochromatic lighting or lighting with light fluxes with a specially selected spectral composition [73]. A feature of LEDs is the ability to produce a high-intensity luminous flux, which is similar in spectral composition to the sun. For example, a white LED has two pronounced maxima of approximately the same intensity, falling on the part of the spectrum with a wavelength of 450 nm and 550–650 nm [74]. It should also be noted that the use of LED lighting can significantly reduce the energy intensity of production [71,75,76].

## 3. Influence of Lighting Intensity

### 3.1. Optimal and Photoinhibiting Light Intensity

Algae grown under various light intensities show changes in biomass formation rate, pigment and lipid content, fatty acid composition, and ratio. For this purpose, various researchers have studied a fairly wide range of light intensity: from 5 to 3500 µmol photons m^−2^ s^−1^ [31,43,47,53,77,78,79,80].

The optimal values of the light intensity at which the maximum growth rate is observed for different taxonomic groups and species of algae, as our analysis of publications shows, are in the range of 26–400 µmol photons m^−2^ s^−1^ (Table 2). Only a few species show extremely high adaptability to high light levels and thus highly effective photoprotection mechanisms. An example of this is *Dunaliella salina*, some strains of which had maximum growth under lighting of 1000 µmol photons m^−2^ s^−1^ [48].

It is known that not all light energy absorbed by the photosynthetic apparatus of algae can be used for photosynthetic reactions. When the maximum rate of photosynthesis is reached, the excess light flux continues to be absorbed by the cell and this leads to a state of light saturation of photosynthesis. At the same time, there is a decrease in the rate of photosynthesis and algae growth [79,86,94,95].

Lighting above which growth slows down, according to Edwards et al. [15], does not differ among the taxonomic groups of marine phytoplankton and varies widely from 20 to 1000 µmol photons m^−2^ s^−1^. Some species have low sensitivity to increased lighting and photoinhibition does not appear for a long time; others, on the contrary, have a very small range of optimal lighting. As a result, the total productivity of phytoplankton has a certain stabilization under variable lighting conditions. However, at the level of individual taxa, if the rate of photodamage exceeds the capabilities of protective mechanisms, a decrease in algal growth is observed. Different species show different resistance to photodamage. For example, in *Phaeodactylum tricornutum*, *Chlorella vulgaris*, and *Tetradesmus obliquus* (=*Scenedesmus obliquus*), saturation and subsequent photoinhibition were observed when exposed to light above 100 and 150 µmol photons m^−2^ s^−1^, respectively [81,86], and no photoinhibition was observed in *Microchloropsis salina* (=*Nannochloropsis salina*), even at 850 µmol photons m^−2^ s^−1^ [43]. In *Chlorella* sp. 800, a continuous increase in biomass productivity was noted without the manifestation of photoinhibition from 0.423 g L^−1^ day^−1^ at 200 µmol photons m^−2^ s^−1^, to 0.475 g L^−1^ day^−1^ at 300 µmol photons m^−2^ s^−1^, 0.62 g L^−1^ day^−1^ at 400 µmol photons m^−2^ s^−1^ and 0.707 g L^−1^ day^−1^ at 500 µmol photons m^−2^ s^−1^ [96]. Photoinhibition was not observed under lighting of 1800 µmol photons m^−2^ s^−1^ in alpine snow alga *Chlamydomonas nivalis* [30].

Recent studies have shown that the strains *Chlamydomonas reinhardtii* cc124 and *Chlorella ohadii*, which are able to grow under very strong lighting—3000 and 3500 µmol photons m^−2^ s^−1^, respectively, exhibit high resistance to photoinhibition [31,80]. The strain *Chlorella ohadii* was isolated from a biological sand crust from the Sinai Desert in the northwestern Negev, Israel [31], and *Chlamydomonas reinhardtii* cc124 is a laboratory strain widely used as a wild type. It should be noted that this strain carries the *agg1* mutation, which causes cells to float away from the light source (negative phototaxis), in contrast to cells of other wild type strains that float towards the light source (positive phototaxis) [97], requiring further study from the standpoint of high resistance to photoinhibition.

### 3.2. Effect of Light Intensity on Lipid Accumulation and Productivity

An increase in light intensity leads to a shift in metabolic processes in algal cells. A number of studies have demonstrated that intense light leads to increased lipid synthesis and lipid excess accumulation. For example, in *Tetradesmus obliquus*, under lighting of 10 µmol photons m^−2^ s^−1^, lipid synthesis does not occur at all, and with increasing lighting, they begin to be produced, and at 200 µmol photons m^−2^ s^−1^, their number reaches maximum values—45.31% of dry biomass [53]. However, a further increase in the light intensity (up to 1000 µmol photons m^−2^ s^−1^) led to a decrease in the lipid content to 38.2% of dry biomass.

The light intensity that stimulates lipid synthesis in different types of microalgae is different (Table 3). The results obtained in the study of various *Chlorella* species and strains are quite indicative in this regard [18,44,96,98].

Under conditions of increased lighting, in the composition of total lipids in many microalgae species, an increase in the content of neutral lipids (NLs)—triacylglyceride (TAG), is observed [44,88]. High light conditions caused the formation of NLs in *Scenedesmus* sp., *Nannochloropsis* sp., *Botryococcus braunii, Isochrysis galbana, Phaeodactylum tricornutum* [47,99,100]. *Chlorella* sp. at 400 µmol photon m^−2^ s^−1^ had NLs 3 times higher than under lighting of 40 µmol photon m^−2^ s^−1^, and the change in the number of membrane lipids had the opposite tendency [44]. However, this is not a common regularity. For example, *Rhodomonas* sp. with an increase in illumination from 150 to 600 µmol photon m^−2^ s^−1^ increases the total fatty acid (TFA) content in membrane structures [84]. Nogueira et al. [99], having studied the effect of light intensity of 50, 300, and 600 µmol photons m^−2^ s^−1^ on the TAG content in *Phaeodactylum tricornutum*, recorded their maximum content under lighting of 600 µmol photons m^−2^ s^−1^. When illuminated with 50 µmol photons m^−2^ s^−1^, the TAG content reduced almost by half. Completely different results were obtained for *Phaeodactylum tricornutum* by Remmers et al. [81] when studying the effect of light intensity in the range 60–750 µmol photons m^−2^ s^−1^. The optimal lighting for TAG production was 60 µmol photons m^−2^ s^−1^. 

In general, as various studies show, the optimal light intensity for the highest lipid content and productivity is not the same for different taxa of microalgae. The composition of lipids itself is not the same within both large taxonomic groups and at the level of strains of the same species [12]. This issue has to be taken into account in further studies on the impact of lighting conditions on the content and production of lipids by microalgae. It should also be mentioned that stress in different taxonomic groups of microalgae can cause a shift in metabolic processes not only in terms of the accumulation of lipids but also in terms of other spare compounds, for example, starch [41].

### 3.3. Changes in the Amount and Composition of Fatty Acids in Response to the Action of Light with Different Intensities

Fatty acids in microalgae cells are in a free state or are part of neutral and polar lipids and fulfill both structural and functional roles [12]. Polar lipids (phospholipids, glycolipids, and betaine lipids) and associated fatty acids are structural components of cell membranes and play an important role in the organization of the light-harvesting complex of microalgae [101]. The fatty acid composition of lipids exhibits taxonomic specificity and also depends on environmental conditions [12]. Changes in light intensity are accompanied by restructuring of the fatty acid composition in microalgae [102]. Previous studies have shown that fatty acids can affect the properties of photosynthetically active membranes, and thereby participate in the biochemical adaptation of microalgae to changes in lighting conditions (for more details, see Wacker et al. [101]. Saturated (SFA) and unsaturated (UFA) fatty acids are believed to have different effects on photosystem II recovery after exposure to high intensity light, with PUFAs being reported to be easy targets for ROS [103]. It is also known that PUFAs are involved in the regulation of membrane fluidity, and a decrease in their number increases membrane rigidity [94]. The effect of light stress on fatty acid composition in comparison with normal growth conditions for some microalgae is summarized in Table 4.

An increase in the light intensity up to 500 µmol photons m^−2^ s^−1^ during the cultivation of *Chlorella* sp. 800 resulted in a 26% increase in fatty acid production compared to cultivation at 200 µmol photons m^−2^ s^−1^ [96]. A similar result of an increase in the content of TFAs and, in addition, arachidonic acid (ARA) were previously observed in *Lobosphaera incisa* (=*Parietochloris incisa*) under lighting of 400 µmol photons m^−2^ s^−1^ [88]. According to the researchers, this is due to the large increase in biomass under such lighting. A high content of directly ARA in *Lobosphaera incisa* cultures was achieved with the simultaneous use of nitrogen starvation and exposure to light of 2000 µmol photons m^−2^ s^−1^ [77].

**Table 3:** Influence of different light intensity on the content and productivity of lipids in microalgae.

Investigated Light Intensity, μmol photons m^−2^ s^−1^ (lx)	Optimal Light Intensity for Lipid Synthesis, μmol photons m^−2^ s^−1^	Species and Strain	Class	Lipid Content, %	Lipid Productivity, mg L^−1^ day^−1^	References
130, 260, 390, 520	520	*Chlorella vulgaris*	Trebouxiophyceae	22.24	19	[18]
600		*Chlorella vulgaris* NIES-2170	Trebouxiophyceae		290	[98]
600		*Chloroidium viscosum* SAG 2338 (=*Chlorella viscosa*)	Trebouxiophyceae		81	[98]
600		*Chlorella sorokiniana* NIES-2169	Trebouxiophyceae		39	[98]
600		*Graesiella emersonii* NIES-2151 (=*Chlorella emersonii*)	Trebouxiophyceae		230	[98]
40, 200, 400	400	*Chlorella* sp.	Trebouxiophyceae	71.66 (NL)	51.36 (NL)	[44]
200		*Chlorella* sp. 589	Trebouxiophyceae	30.2	102	[96]
200		*Chloroidium saccharophilum* 477 (=*Chlorella saccharophila*)	Trebouxiophyceae	27.6	72,5	[96]
200		*Chlorella* sp. 800	Trebouxiophyceae	24.4	121	[96]
600		*Parachlorella beijerinckii* SAG 2046	Trebouxiophyceae		190	[98]
600		*Parachlorella kessleri* NIES-2152	Trebouxiophyceae		330	[98]
600		*Parachlorella kessleri* NIES-2159	Trebouxiophyceae		210	[98]
600		*Parachlorella kessleri* CCALA 25	Trebouxiophyceae		200	[98]
50, 250, 400	400	*Scenedesmus* sp. 11-1	Trebouxiophyceae	41.1		[104]
10–1000	200	*Tetradesmus obliquus*	Trebouxiophyceae	45.31		[53]
405		*Choricystis* sp. LBB13-AL045	Trebouxiophyceae		46.13	[105]
200		*Botryococcus braunii* KMITL 2	Trebouxiophyceae	54.69		[106]
84, 133, 182	182	*Tetraselmis* sp. V_2_	Chlorodendrophyceae	49.0		[89]
120		*Tetraselmis suecica*	Chlorophyceae	26.1	47.3	[107]
100–500	300	*Chlamydomonas* sp. JSC4	Chlorophyceae	43.1	312	[108]
200–800	500	*Ettlia* sp. YC001	Chlorophyceae		420	[49]
100, 200	200	*Dunaliella salina* Y6	Chlorophyceae	14.5		[79]
90–300	300	*Haematococcus lacustris* (=*Haematococcus pluvialis*)	Chlorophyceae	32.99		[109]
40, 200, 400	400	*Monoraphidium dybowskii* Y2	Chlorophyceae	60.65 (NL)	49.71 (NL)	[44]
50, 300, 600	600	*Isochrysis galbana*	Coccolithophyceae	31.71	21.67	[99]
50, 300, 600	600	*Phaeodactylum tricornutum*	Bacillariophyceae	39.53 (TAG)	31.39 (TAG)	[99]
60–750	60	*Phaeodactylum tricornutum*	Bacillariophyceae		112 (TAG)	[81]
170, 700	700	*Nannochloropsis* sp.	Eustigmatophyceae	47 (TAG)	360 (TAG)	[47]
50, 100, 200	100	*Nannochloropsis* sp.	Eustigmatophyceae	31.3		[75]
(3000, 4000, 5000)	5000	*Arthrospira platensis* (=*Spirulina platensis*)	Cyanophyceae	35.8		[50]

**Table 4:** The effect of light stress on fatty acid composition of different microalgae.

Investigated Light Intensity, μmol photons m^−2^ s^−1^	Effective Light Intensity for Fatty Acid Synthesis, μmol photons m^−2^ s^−1^	Species	TFA	SFA	MUFA	PUFA		References
						Total PUFA	Individual Fatty Acid	
20, 100, 340	20	*Diacronema lutheri* (=*Pavlova lutheri*)				↑	↑EPA	[110]
37.5, 62.5, 100	37.5	*Chlorella vulgaris*			↑	↑		[111]
37.5, 62.5, 100	100	*Chlorella vulgaris*		↑				[111]
150, 750	150	*Phaeodactylum tricornutum*				↑	↑EPA	[102]
50, 125, 300	300	*Chlorella vulgaris*, *Tetradesmus obliquus*			↑18:1		↓18:3	[86]
50, 125, 325	325	*Isochrysis galbana*		↑16:0	↑18:0	↓	↓LA, ↓ALA, ↓SDA	[85]
20, 100, 340	340	*Diacronema lutheri*		↑		↑	↑DHA	[110]
35, 200, 400	400	*Lobosphaera incisa*	↑			↑	↑ARA	[88]
30, 100, 400	400	*Entomoneis paludosa*, *Nitzschia alexandrina*, *Staurosira* sp.				↓	↓ARA, ↓EPA	[95]
200, 500	500	*Chlorella* sp.	↑					[96]
80, 500	500	*Nostoc carneum* (=*Nostoc spongiaeforme*), *Phormidium corium*	↓					[112]
60,195, 330, 465, 600	600	*Rhodomonas* sp.				↑	↑EPA, ↑DHA	[84]
150, 750	750	*Phaeodactylum tricornutum*		↑		↓		[102]
5, 25, 50, 100, 250, 850	850	*Microchloropsis salina*	↑	↑			↓EPA	[43]
250, 2000	2000	*Lobosphaera incisa*					↑ARA	[77]

Down arrow ↓ means decrease, up arrow ↑ means increase.

Many studies have shown that intense light promotes the accumulation of SFAs in cells and a decrease in UFAs and PUFAs as well. A decrease in the production of PUFAs, ARA, and eicosapentaenoic acid (EPA) in *Entomoneis paludosa* NCC18.2, *Nitzschia alexandrina* NCC33, and *Staurosira* sp. NCC182 with an increase in light intensity from 30 to 400 µmol photons m^−2^ s^−1^ was recorded in studies by Cointet et al. [95]. They concluded that low light and no nitrogen restrictions resulted in better photosynthetic energy-saving potential of the three strains, and therefore a more profitable approach. In *Phaeodactylum tricornutum*, light of 150 µmol photons m^−2^ s^−1^ led to an increase in the production of PUFAs (16.59%), including EPA (5.72%). In contrast, irradiation with 750 µmol photons m^−2^ s^−1^ led to an increase in SFAs and a decrease in PUFA concentrations in the fatty acid distribution [102].

In the green microalgae *Chlorella* vulgaris, *Tetradesmus obliquus*, an increase in light intensity from 50 to 300 µmol photons m^−2^ s^−1^ led to a decrease in the linolenic 18:3 acid content and an increase in the oleic 18:1 acid content, which is important for improving the quality of biodiesel in its production from microalgae [86]. Khoeyi et al. [111] previously reported that in *Chlorella* *vulgaris*, the maximum percentage of total SFAs (33.38%) was recorded at 100 µmol photons m^−2^ s^−1^ and a photoperiod 16:8 h (light:dark), while the maximum production of monounsaturated fatty acids (MUFAs) 15.93% and PUFAs 27.40% were recorded at 37.5 µmol photons m^−2^ s^−1^ and photoperiod 8:16 h (light:dark).

*Microchloropsis salina* showed a sharp increase in TFAs containing increased amounts of palmitic 16:0 and palmitoleic 16:1 acids under bright light, indicating a possible accumulation of TAG due to photooxidative stress. Lower lighting results in an increase in the relative content of UFAs [43]. Mishra et al. [85] observed in *Isochrysis galbana* the replacement of the accumulation of one fatty acid group, including myristic 14:0, linoleic 18:2 (LA), α-linolenic 18:3 (ALA), and stearidonic 18:4 (SDA) acids, with a second fatty acid group, including palmitic 16:0, and oleic 18:1 acids, under conditions of nitrogen limitation and high light intensity. In *Diacronema lutheri* (=*Pavlova lutheri*), PUFA content, especially EPA, were significantly higher in low light, and levels of SFAs and docosahexaenoic acid (DHA) were significantly higher in bright light [110].

Fatty acid desaturation is considered to be an important factor in increasing the resistance of *Synechococcus* sp. PCC 7942 to strong light, especially at low temperatures [113]. Bhandari and Sharma [112] observed a decrease in the content of phospholipids and glycolipids in the cyanobacteria *Nostoc spongiiforme* and *Phormidium corium* when exposed to strong light 500 µmol photons m^−2^ s^−1^. At the same time, the level of UFAs in total lipids and glycolipids remained unchanged in both species, but the level of SFAs decreased. This slightly changed the ratio in favor of UFAs at high light. No effect on the number of double bonds in fatty acid molecules when the light intensity changes from 200 to 500 was noted for *Chlorella* sp. 800 [96].

Thus, the available data on changes in the content of SFA and UFA in response to changes in light intensity are contradictory. It has been suggested that at high light intensity, microalgal cells use excess energy to produce storage lipids, mainly SFA and MUFA, which are usually involved in the formation of thylakoid membranes, and at low light levels, the PUFA content increases, mainly SDA, EPA, and octadecapentaenoic 18:5 omega-3 acids. The increase in the content of MUFAs (including oleic 18:1 acid) is associated with its central role in the PUFA synthesis system [85]. It is also shown that the active functioning of photosystem I, caused by a change in the light intensity, is accompanied by an increase in omega-3 desaturation of fatty acids. There is a correlation between the formation of omega-3 fatty acids in monogalactosyldiacylglycerols and the formation of reaction centers of photosystems, as well as between the biosynthesis of omega-6 fatty acids and the formation of light-harvesting complexes of photosystems. Omega-3 desaturation of fatty acids can be considered as one of the quick adaptive responses to changes in light intensity; it does not always lead to an increase in the total lipid unsaturation index and can be associated with the “transition states” of photosystems [114].

It is reported that the change in the composition of fatty acids in response to the action of intense illumination may be due to the specificity of the fatty acid composition of individual taxa of microalgae. It may also be related to differences in the strategy of light acclimatization of different species [101].

### 3.4. Production of Carotenoids When Changing Light Intensity

Carotenoids in cells are both bound and free. The pathways of biosynthesis and transformation of carotenoids in microalgal cells are being thoroughly studied and information about this is available in a number of works [20,64,68]. Carotenoids play an important role in the photosynthesis processes of microalgae, as additional pigments, as well as in the mechanisms of photoprotection when exposed to high intensity light. Photoprotective mechanisms involving carotenoids in algae are quite diverse and have been discussed in detail in a number of recent works [67,70,115,116].

One of the main mechanisms of photoprotection is based on the ability of some xanthophylls to participate in reversible light-dependent reactions, in which the excess excitation energy of chlorophylls is dissipated in the form of heat. This mechanism is called non-photochemical quenching and is well documented [15,63,117]. In algae, the most common xanthophyll cycles are violaxanthin and diadinoxanthin. The violaxanthin cycle is based on the mutual transformation of xanthophylls of violaxanthin, antheraxanthin, and zeaxanthin. In bright light, violaxanthin is converted to zeaxanthin via the antheraxanthin intermediate, while in low light, zeaxanthin is converted back to violaxanthin. The diadinoxanthin cycle includes interconversions of acetylenic (having one triple bond) xanthophylls. With excessive light, diadinoxanthin is converted to diatoxanthin, and with low light, the opposite reaction is observed [115,118,119].

In addition to non-photochemical quenching, carotenoids can be involved in the chemical or physical suppression of ^1^O_2_, which is formed under conditions of high light intensity. It is believed that β-carotene quenches ^1^O_2_ in the photosystem II reaction center, which is the main site of ^1^O_2_ formation [15]. Carotenoids in cells can also act as filters that reduce the amount of light reaching the photosynthetic apparatus [67]. It is reported that some carotenoids can accumulate in microalgae cells only under long-term exposure to strong light [64].

The ability of microalgae to accumulate carotenoids in large quantities makes them valuable objects for biotechnological production. On the world market, there is an increase in interest in carotenoids from natural sources, including microalgae [11]. Microalgae such as *Dunaliella salina*, *Haematococcus lacustris* (=*Haematococcus pluvialis*), and some *Chlorella* are rich sources of carotenoids [37,38,48,66]. In microalgae capable of both autotrophic and heterotrophic growth (for example, *Haematococcus lacustris* and *Chromochloris zofingiensis*, formerly known as *Chlorella zofingiensis*), heterotrophic conditions can significantly increase the productivity of biomass, but they cannot provide large amounts of pigments, including commercially valuable ones [120]. Exposure to high-intensity lighting increases carotenoid production in *Haematococcus lacustris* [37,121], *Dunaliella salina* [48,79], *Chromochloris zofingiensis* [122], *Tetradesmus* *obliquus* [123], *Nostoc calcicola* [124], etc. (Table 5). The composition of carotenoids in different microalgae species is not the same [21,68]. These species-specific features are reflected in the composition of carotenoids that accumulate under the action of light stress.

Astaxanthin and β-carotene are most interesting among carotenoids in the world market, and the demand for lutein and zeaxanthin is also growing [11,13]. β-carotene is the most widespread carotenoid in microalgae. It is the primary carotenoid. It takes part in photosynthesis and photoprotection. β-carotene is a biosynthetic precursor of xanthophylls such as zeaxanthin, antheraxanthin, violaxanthin, and neoxanthin. Large amounts of β-carotene, as well as lutein and zeaxanthin, are produced in *Dunaliella salina* [48]. It is a known fact that the production of these carotenoids in *Dunaliella salina* increases with increasing light intensity. Thus, an increase in lighting from 100 to 200 µmol photons m^−2^ s^−1^ increased the accumulation of β-carotene in *Dunaliella salina* Y6 by 31.5% [79]. In other studies, *Dunaliella* strains (DF15, DF17, DF40, UTEX 253) demonstrated an increase in β-carotene productivity under lighting of more than 200 µmol photons m^−2^ s^−1^ and maximum productivity values (up to 3.5 mg L^−1^ day^−1^) were noted at 1500 µmol photons m^−2^ s^−1^ [48].

Astaxanthin is a secondary carotenoid belonging to the xanthophyll group. Its precursor is β-carotene [129]. Astaxanthin has 13 conjugated double bonds arranged as alternating single-double bonds. This configuration confers astaxanthin strong antioxidant properties thereby scavenging ROS and neutralizing free radicals. It is reported that when exposed to stressful conditions, some microalgae synthesize and accumulate astaxanthin in significant quantities [68]. Known astaxanthin producers are *Haematococcus lacustris* [37,121,130,131], and *Chromochloris zofingiensis* [122,132,133]. Smaller amounts of astaxanthin were found in *Neochloris wimmeri, Scenedesmus vacuolatus* [125], and *Chlamydomonas nivalis* [134].

High light intensity is one of the most effective factors for stimulating astaxanthin synthesis in microalgae [37]. There are positive results of an increase in the productivity of astaxanthin when exposed to high intensity light (100 and 400 µmol photons m^−2^ s^−1^) on heterotrophically grown cultures of *Haematococcus lacustris* NIES-14, which have previously undergone the acclimatization procedure with moderate light intensity (25 and 50 µmol photons m^−2^ s^−1^) [22]. Using this strategy, the productivity of astaxanthin was obtained up to 10.5 mg L^−1^ day^−1^, and the most efficient light was 100 µmol photons m^−2^ s^−1^. In other studies, *Haematococcus lacustris* JNU35 achieved an astaxanthin productivity of 18.1 mg L^−1^ day^−1^ using 150 µmol photons m^−2^ s^−1^ and then 300 µmol photons m^−2^ s^−1^ lighting [135]. A similar strategy of stepped light changing from 50 µmol photons m^−2^ s^−1^ to 400 µmol photons m^−2^ s^−1^ was applied to *Chromochloris zofingiensis* ATCC 30412 and also increased astaxanthin synthesis to 2.7 mg g^−1^ of dry biomass and 9.9 mg L^−1^ day^−1^, respectively. The results obtained under autotrophic conditions were 4.0 and 2.5 times higher than those obtained under heterotrophic conditions [122].

Pigment α-carotene is less common than β-carotene. It is known that in the pathways of carotenoid conversion in algal cells, α-carotene is a precursor of lutein [64]. The content of lutein in cells is affected by high light intensity. It is believed to be involved in the quenching of excited forms of chlorophyll and prevent the formation of ROS [119]. In *Tetraselmis* sp. CTP4 β-carotene content was maximal at a light intensity of 33 µmol photons m^−2^ s^−1^, while the lutein content increased 1.5 times at higher light intensities—170 and 280 µmol photons m^−2^ s^−1^ [127]. Light of 170 µmol photons m^−2^ s^−1^ for 2 days contributed to the high content in *Tetraselmis* sp. CTP4 of both lutein and β-carotene: 3.17±0.18 and 3.21±0.18 mg g^−1^ of dry biomass, respectively [127]. In *Auxenochlorella protothecoides*, the highest productivity of lutein in culture with a heterotrophic-photoautotrophic transition reached 12.36 mg L^−1^ day^−1^ on the seventh day. The light intensity at the autotrophic stage of cultivation corresponded to 4000 lx [120].

Thus, an increase in light intensity, taking into account species-specific features, can be used to stimulate the synthesis of carotenoids in microalgae, including β-carotene, lutein and astaxanthin.

## 4. Influence of Spectral Composition of Light

Pigments included in the structure of photosynthetic complexes of microalgae absorb photons from different parts of the light spectrum. Different microalgae taxonomic groups have different compositions of photosynthetic pigments and, accordingly, differ in the spectrum breadth that can be used in photosynthesis. For the maximum speed of photosynthesis, the spectral composition of light should be optimal for the pigment composition of the photosynthetic complex of a particular type of microalgae [39].

The use of LEDs as a light source allows both monochromatic lighting of microalgae cultures and simultaneous lighting with a light flux of a certain spectral composition. It is reported that the use of this technology can improve the efficiency of photochemical reactions [73] and the profitability of production [71,76]. It is also extremely important that the light flux of a special spectral composition can change metabolic processes in microalgae cells, shifting them towards the predominant accumulation of proteins, carbohydrates, or lipids [39]. Jungandreas et al. [136] reported that the transition from red to blue light in *Phaeodactylum tricornutum* increases the lipid content, and the reverse transition from blue to red light promotes the synthesis of carbohydrates. Blue light increases carbohydrate content, and the addition of green light can increase protein content in *Arthrospira platensis* (=*Spirulina platensis*) [137].

Various studies have examined the effects of red, yellow, green, and blue light on microalgae compared to each other and white light. The effect of monochromatic and mixed lighting on the growth of microalgae, lipid accumulation, and the composition of fatty acids and carotenoids was also studied (Table 6).

Red light stimulated biomass formation in *Phaeodactylum tricornutum* [72], red and blue light in *Scenedesmus* sp. [142], red and yellow in *Nannochloropsis* sp. [143]. Okumura et al. [76] found that monochromatic blue and mixed red-green-blue LEDs contributed to the formation of the highest biomass in *Botryococcus braunii* (0.22 and 0.39 mg mL^−1^ d^−1^, respectively) and the highest relative growth rate (9.20 ± 2.16 and 9.47 ± 2.67 µg mL^−1^ d^−1^, respectively). Good results in increasing biomass are achieved with the simultaneous use of blue and red LEDs in *Chlamydomonas reinhardtii* [144], *Arthrospira platensis* [137], *Auxenochlorella pyrenoidosa*, =*Chlorella pyrenoidosa* [145]. In *Arthrospira platensis*, the highest biomass formation rates were observed under red lighting, and the lowest under blue lighting [91,146]. Somewhat different results were obtained by Prates et al. [147] in *Spirulina* sp. LEB 18. Blue, green, red, and white LEDs increased biomass productivity and maximum specific growth rate in *Spirulina* sp. LEB 18; the maximum values were achieved with red LEDs, and with green ones, the highest concentration of phycocyanin was noted [147].

In *Parachlorella kessleri* UTEX 398, red light stimulates cell formation, while blue light stimulates cell growth [148]. The authors believe this can be used in the *Parachlorella kessleri* production run by first applying red light to obtain the desired cell concentration and then switching to blue light to increase cell size.

An increase in biomass productivity and lipid production in *Tetraselmis* sp., *Nannochloropsis* sp., *Chlorella vulgaris*, *Isochrysis galbana* was achieved with the use of blue LEDs [71,75,141,149,150]. Red light stimulates lipid formation in *Botryococcus braunii* [138], *Nannochloropsis* sp. [143], red and blue—in *Arthrospira platensis*, *Chlorella vulgaris*, *Tetradesmus obliquus*, *Diacronema lutheri* [39,139]. In *Rhodomonas* sp., blue and red lighting decreases biomass production, while green lighting increases it [151]. This is explained by the more efficient use of light energy due to the increased absorption of green and blue light waves by phycoerythrin, chlorophyll, and carotenoids of *Rhodomonas* sp. Green light has a stimulating effect on lipid synthesis in *Porphyridium purpureum*, =*Porphyridium cruentum* [139].

When *Chlorella vulgaris* was grown in blue light at three different light intensities (100, 200, and 300 µmol photons m^−2^ s^−1^), the maximum lipid content (23.5%) was obtained by lighting of 200 µmol photons m^−2^ s^−1^ with blue light and photoperiod 12:12 h light:dark [75]. When using intermittent blue light with a cycle of 50 µs/50 µs (light:dark), the lipid content in *Isochrysis galbana* reached 155 mg L^−1^, and with constant white light with a photon flux density of 104 µmol photons m^−2^ s^−1^ with a cycle 12:12 h (light:dark)—98 mg L^−1^. Culturing under intermittent blue light also led to the highest accumulation of DHA in *Isochrysis galbana* [141].

Red light in *Phaeodactylum tricornutum* stimulated the formation of PUFAs, and the amount of MUFAs decreased [72]. The use of red LEDs led to an increase in the content of EPA in the biomass of *Tetraselmis suecica* [24], EPA and DHA in the biomass of *Porphyridium purpureum*, while in the biomass of *Diacronema lutheri*, *Chlorella vulgaris*, an increase in the content of EPA and DHA occurred during lighting with yellow and green LEDs [139]. Blue light in *Chlorella vulgaris*, *Auxenochlorella pyrenoidosa*, *Scenedesmus quadricauda*, *Tetradesmus obliquus* stimulated the formation of PUFAs and directly ALA [140].

A high content of carotenoids was obtained in the presence of green LED lighting in *Arthrospira platensis* and yellow LED lighting in *Chlorella vulgaris* and *Tetradesmus obliquus*. The highest content of carotenoids was in *Arthrospira platensis* (6.1 mg g^−1^). The second largest result was recorded, respectively, with yellow and green LED lighting [39].

The results obtained in various studies indicate that the use of red light, as well as blue, improves the biomass productivity of microalgae of various taxonomic groups. It is known that the absorption maxima of chlorophylls are in the red and blue regions of the light spectrum, which ensures the most intense photosynthesis when using red and blue rays. Carotenoids, as additional pigments, actively absorb the blue-violet part of the spectrum and transfer the absorbed energy to chlorophyll. The significance of blue rays during the growth of microalgae and their productivity is enhanced by carotenoids.

## 5. Influence of Duration and Frequency of Lighting

The growth and metabolism of photoautotrophic microalgae is greatly influenced by the duration and frequency of lighting [84]. When cultivating microalgae, both continuous lighting and alternation of light and dark cycles, as well as light flashes or pulsed lighting, are used [46]. It is assumed that the use of lighting systems with dark cycles can increase the productivity of microalgae by increasing the efficiency of light absorption and reduce energy consumption, which is important for increasing the profitability of biotechnological production of microalgae. In addition, it is reported that continuous lighting can initiate photoinhibition processes, while the use of dark periods, including short-term ones, can help restore damage to photosystems [54].

By changing the photoperiod duration and varying the light intensity, it is possible to change the biochemical composition of microalgae. For example, *Nostoc calcicola* at a light intensity of 21 µmol photons m^−2^ s^−1^ and a photoperiod of 8:16 light:darkness accumulated proteins and phycobiliproteins, and at a light intensity of 63 µmol photons m^−2^ s^−1^ and longer illumination (16:8 light:dark)—carbohydrates and carotenoids [124].

An increase of *Rhodomonas* sp. biomass yield by 22% was achieved at a cycle 16:8 h (light:dark) and lighting of 600 µmol photons m^−2^ s^−1^ compared to constant lighting of 150 µmol photons m^−2^ s^−1^ [84]. Also, the realization of a cycle light:dark made it possible to increase the content of TFAs in the biomass of *Rhodomonas* sp. by 19% and EPA + DHA content by almost 14% compared to the same levels for constant lighting conditions. The studies have shown that the use of light and dark cycles allows choosing the most appropriate time for collecting biomass. Thus, the best time to obtain biomass of *Rhodomonas* sp. with maximum EPA + DHA is before the start of the light cycle, and if maximum TFA content is required, the best time is between 5–9 h after the start of lighting.

The duration of the light period affects not only the growth of microalgae, but also the lipid content. For example, growing *Nannochloropsis* sp. in three different cycles of the photoperiod (24:0 h, 18:6 h, and 12:12 h of light:dark) and different lighting intensities (50, 100, and 200 µmol photons m^−2^ s^−1^) showed that a light intensity of 100 µmol photons m^−2^ s^−1^ and a photoperiod 18:6 h (light:dark) was the most productive and produced a lipid content of 31.3% [75]. Increased biomass production and lipid content in *Nannochloropsis* sp. was obtained using a flashing light with a 45:15 min (light:dark) cycle [54].

Manipulations with the photoperiod duration and the light intensity make it possible to alter the production of carotenoids by microalgae. For example, in *Nostoc calcicola*, an increase in the light intensity from 21 to 63 µmol photons m^−2^ s^−1^ and the photoperiod duration from 8:16 to 16:8 h (light:dark) led to an increase in the content of carotenoids [124]. High light intensity and prolonged photoperiods were favorable for increasing the concentration of astaxanthin in *Haematococcus lacustris* cultures [37].

Using light of different wavelengths and varying the photoperiod duration, changes in the production characteristics of microalgae are also achieved. For example, white light with a cycle 24:0 h (light:dark) is better than blue and red light and a cycle 12:12 h (light:dark) for the growth of *Haematococcus* *lacustris*, as well as lipid and astaxanthin production [38].

Another strategy for altering microalgae growth and lipid production is the use of pulsed light. It has been demonstrated that even strong light (for example, 1000–1200 µmol photons m^−2^ s^−1^) exceeding the saturation point of photosynthesis can be used for the microalgae growth [45]. In experiments with pulsed light, cells not only were able to avoid photodamage, but also used the energy from the pulses with the same efficiency as with dim continuous light. Moreover, it was found that the intensity of light flashes is not very important for growth. The results of the growth of *Microchloropsis salina* under light flashes of 1200 and 350 µmol photons m^−2^ s^−1^ were similar. The authors’ observations confirmed the importance of such a parameter as the duration of a light pulse. The optimal duration of light pulses was found to be around 10 ms. It is known that after photon absorption by the photosystem, 1–15 ms are needed to reboot the system before it is ready to receive the next photon. Under short illumination, even with strong light, most photons are used for photosynthesis and do not lead to the formation of ROS, which then causes photoinhibition [45]. Cultivation of *Isochrysis galbana* at a flux density of 52 µmol photons m^−2^ s^−1^ using white, red and blue light at a frequency of 10,000 Hz in the form of cycles 50 µs/50 µs (light:dark) allowed to find out that pulsed white light contributes to better growth than continuous white light, and lipid production was higher with intermittent blue light [141]. However, the use of pulsed light does not always lead to the stimulation of microalgae growth. It was noted that illumination conditions of 1200 photons m^−2^ s^−1^ at 10 Hz and 350 photons m^−2^ s^−1^ at 30 Hz lead to the greatest growth of *Microchloropsis salina*, and light 1200 photons m^−2^ s^−1^ at 1–5 Hz to growth inhibition [45].

## 6. Simultaneous Use of Light and Other Stresses

It is well documented that exposure of microalgae to more than one stress at the same time, for example, high light intensity and high salinity or high light intensity and lack of nitrogen, phosphorus, can contribute to a stronger effect on the productivity of algae and their ability to accumulate lipids, valuable carotenoids [37,95,106].

The combination of mild salt stress (50 mM NaCl) and light exposure resulted in greater lipid production in *Chlamydomonas reinhardtii* CC124 compared to heterotrophic conditions. However, a high concentration of NaCl (200 mM) caused irreversible damage to *Chlamydomonas reinhardtii* CC124 cells [94]. An increased susceptibility to photoinhibition was also observed in *Arthrospira platensis* under simultaneous exposure to light and salt stress [152]. An increase in the light intensity from 100 to 200 µmol photons m^−2^ s^−1^ was accompanied by an increase in the growth rate of *Arthrospira platensis*, while the addition of NaCl in the amount of 0.5 and 0.75 M did not increase the growth rate with increasing lighting. This indicated a reduced ability of *Arthrospira platensis* to use light energy under conditions of increased NaCl concentration.

Bright light enhances salt stress-induced astaxanthin and lipid biosynthesis in the green alga *Chromochloris zofingiensis* [153]. The best condition for lipid and astaxanthin production in *Haematococcus* *lacustris* was high intensity light and nitrogen starvation [38]. Other studies found that phosphate starvation and light stress also increased astaxanthin productivity in *Haematococcus* *lacustris*. Limiting phosphates to 41 mg L^−1^ was most effective; 0.25× of the standard BBM scale and continuous light stress (5000 lx) [37].

Pelah et al. [69] believe that salt stress can replace light stress with respect to the induction of carotenoid production: *Chromochloris zofingiensis* cells grown in low light and subjected to salt stress and low nitrogen stress accumulated more secondary carotenoids than cells growing in high light and low nitrogen. At the same time, the best conditions for the accumulation of canthaxanthin in *Chromochloris zofingiensis* were salt stress and low lighting, and for the accumulation of astaxanthin, high lighting was required. Li et al. [132] suggested that stresses due to strong light and NaCl can stimulate the generation of various ROS, which, in turn, act on certain carotenogenic genes responsible for triggering enhanced carotenoid biosynthesis in *Chromochloris zofingiensis*.

Study of the combined effect of light (60–195–330–465–600 µmol photons m^−2^ s^−1^) and temperature (15–20–25–30 °C) on the production and biomass composition of *Rhodomonas* sp. showed that stable biomass production was observed under all conditions, with the exception of experiments carried out at 30 °C, which led to cell death. The optimal temperature was 22–24 °C and light 110 to 220 µmol photons m^−2^ s^−1^. The fatty acid profile was significantly influenced only by temperature, namely, higher EPA and DHA contents were found at lower temperatures (15 °C), and the maximum production rate of EPA + DHA was obtained at 20 °C and strong light conditions (600 µmol photons m^−2^ s^−1^) [84]. In *Isochrysis galbana*, the combined effect of temperature and light intensity increased the TAG content (from 18.59 to 31.71%) and its accumulation productivity (from 11.76 to 21.67 mg L^−1^ day^−1^) [99].

In general, the sensitivity of algae to lighting conditions changes with the adjustment of the temperature regime. Light limitation lowers the temperature optimum of the species by ~5 °C. There is an indication that the light saturation point changes with temperature and decreases with decreasing temperature [154].

Thus, when assessing the impact of light on the growth of microalgae and their production characteristics, it is necessary to take into account the impact of other parameters: temperature, composition of the cultivation medium, CO_2_ concentration, etc. These issues are discussed in several research works [47,51,69,85]. However, the complexity and ambiguity of the observed dependencies do not allow us to single out general patterns which are applicable for various taxonomic algae groups and various combinations of parameters.

## 7. Conclusions

Light is a prerequisite for the photoautotrophic growth of microalgae. Light intensity, spectral composition, duration, and frequency of lighting can act as an effective tool for regulating the growth of microalgae, changes in metabolism, and induction of compounds valuable from a biochemical point of view. 

The optimal light intensity for the growth of microalgae usually lies in the following range: 26–400 µmol photons m^−2^ s^−1^. Only a few species demonstrate extremely high adaptation to high levels of lighting (up to 3000–3500 µmol photons m^−2^ s^−1^) and, accordingly, highly effective photoprotection mechanisms.

An increase in light intensity leads to increasing the lipid synthesis. For maximum lipid productivity, various species and strains of microalgae need lighting of different intensities: from 60 to 700 µmol photons m^−2^ s^−1^. Strong light facilitates the accumulation of lipid especially TAGs. Probably, this may be as a means to convert excess light to chemical energy to avoid photooxidative damage.

Exposure to high intensity light stimulates the formation of fatty acids and changes in their composition. The fatty acid composition, the ratio of the amount of SFAs and UFAs can affect the properties of membranes, the activity of photosystem I functioning and the processes of photosystem II recovery after exposure to high light intensity. Generally, there is an accumulation of SFAs, as well as MUFAs and a decrease in the number of PUFAs. However, some microalgae under intense lighting conditions can increase the production of ARA, EPA, and DHA. 

High light intensity is one of the most effective factors in stimulating the synthesis of carotenoids, including β-carotene, lutein, and astaxanthin in microalgae. 

The use of a light flux of a special spectral composition changes metabolic processes in microalgae cells, as well as the composition of compounds accumulated by cells. Red as well as blue LED lighting improves the biomass productivity of microalgae of various taxonomic groups. 

Manipulations with the duration of the photoperiod make it possible to increase the microalgae productivity by increasing the efficiency of light absorption. A feature of using pulsed light is the ability to avoid photodamage of microalgae cells and create conditions for the most efficient absorption of light photons during a flash and restoration of photosystems in the period between flashes. This is because under pulsed light, even with strong light, the majority of photons are used for photosynthesis and do not lead to the formation of ROS, which then cause photoinhibition.

The optimal light conditions for photosynthesis, growth, accumulation of lipids, SFAs, MUFAs and PUFAs, carotenes differ both in different taxonomic groups and in different strains of the same species. Moreover, the available data are sometimes contradictory and ambiguous. Taking this into account, as well as the existing developments in this area, there are a number of new tasks for further research:

- unification of approaches to studying the effect of lighting on the growth and productivity of microalgae;
- finding out the optimal lighting conditions for the cultivation of biotechnologically valuable species and strains of microalgae;
- establishing relationships between changes in lighting conditions, including light intensity, spectral composition of light, duration of lighting, etc. and an increase in the synthesis of compounds valuable from a biochemical point of view by microalgae;
- determination of the stimulatory effects of a combination of light stress with other types of stress on the growth and productivity of microalgae, taking into account the synthesis of valuable bioproducts (lipids, fatty acids, carotenoids).

In order to standardize the approaches to the study of the impact of light on the growth and productivity of microalgae, it is recommended not only to provide information on light parameters, cell density in cultures, volume of the medium used, its composition, and CO_2_ concentration in the experimental studies, but also to indicate the origin of strains. When using strains of natural origin, it is important to indicate the peculiarities of the lighting conditions of its habitat. They can be very different both for aquatic organisms and for terrestrial ones growing in open spaces, in caves, in deep soil layers, etc. In the future, this will simplify the analysis of the results obtained and the development of recommendations for biotechnological industries.

## Figures and Tables

**Table 1:** Main pigments of algal photosystems and absorption maxima of light waves.

Pigments	Absorption Maxima in Organic Solvents *, nm	Representatives	References
chlorophyll *a*	420, 660	All algae	[57,59]
chlorophyll *b*	435, 643	Green algae	[59,60]
chlorophyll *c*	445, 625	Heterokontophyta, Haptophyta, Dinophyta, Cryptophyta	[59,61]
chlorophyll *d*	450, 690	Rhodophyta, some Cyanobacteria	[40,59]
chlorophyll *f*	707	Some Cyanobacteria	[40,59,62]
β-carotene	425, 450, 480	Most algae	[57,59]
α-carotene	420, 440, 470	Some algae (Cryptophyta, Haptophyta, Dinophyta, Chrysophyceae), some Cyanobacteria	[58,63]
fucoxanthin	425, 450, 475	Heterokontophyta (Bacillariophyceae Phaeophyceae, Chrysophyceae), Haptophyta	[64]
phycoerythrin	490, 546, 576	Rhodophyta, Cryptophyta, Cyanobacteria	[58,65]
phycocyanin	618	Rhodophyta, Cryptophyta, Cyanobacteria	[58,65]
allophycocyanin	650	Rhodophyta, Cryptophyta, Cyanobacteria	[65]

* Pigment absorption maxima may vary with different solvents [64].

**Table 2:** Optimal light intensity values for the maximum growth rate of algae from different taxonomic groups.

Investigated Light Intensity, μmol photons m^−2^ s^−1^	Optimal Light Intensity for Maximum Growth, μmol photons m^−2^ s^−1^	Maximum Growth Rate (d^−1^)	Species and Strain	Class	References
5, 25, 50, 100, 250, 850	26–55	1.3	*Microchloropsis salina* (=*Nannochloropsis salina*)	Eustigmatophyceae	[43]
60, 100, 250, 500, 750	60–112	*	*Phaeodactylum tricornutum*	Bacillariophyceae	[81]
70, 140, 210	70	*	*Porphyridium purpureum*	Porphyridiophyceae	[82]
10, 20, 40, 60, 80, 100, 120, 140, 160, 180, 200, 220, 240, 260, 300	60–100	0.6–0.7	*Rhodomonas salina*	Cryptophyceae	[83]
60,195,330, 465, 600	110–220	>1.0	*Rhodomonas* sp.	Cryptophyceae	[84]
50, 125, 325	325	1.1	*Isochrysis galbana*	Coccolithophyceae	[85]
10, 50, 150, 200, 350, 1000	150	0.8	*Tetradesmus obliquus* (*=Scenedesmus obliquus*)	Trebouxiophyceae	[53]
50, 150, 300	150	*	*Scenedesmus obliquus*	Trebouxiophyceae	[86]
50, 150, 300	150	*	*Chlorella vulgaris*	Trebouxiophyceae	[86]
150, 300	150	0.77	*Chromochloris zofingiensis* (=*Chlorella zofingiensis)*	Trebouxiophyceae	[87]
35, 200, 400	400	0.2 ^a^	*Lobosphaeraincisa* (=*Parietochloris incisa*)	Trebouxiophyceae	[88]
133, 182	133	*	*Tetraselmis* sp.	Chlorodendrophyceae	[89]
200, 500, 1000, 1500	1000	0.2 (DF15) 0.55 (UTEX 2538)	*Dunaliella salina* (DF15, UTEX 2538)	Chlorophyceae	[48]
200, 500, 1000, 1500	1500	1.3 (CCAP 19/30) 1.05 (DF17) 0.75 (DF40)	*Dunaliella salina* (CCAP 19/30, DF17, DF40)	Chlorophyceae	[48]
20–500	330	1.78	*Arthrospira fusiformis*	Cyanophyceae	[90]
200–700	360	0.26	*Arthrospira fusiformis*	Cyanophyceae	[91]
40, 160	160	0.491	*Phormidium* sp.	Cyanophyceae	[92]
75, 100, 150, 500, 660, 750	660	2.14 ^b^	*Synechococcus* sp. PCC 11901	Cyanophyceae	[93]
75, 100, 150, 500, 660, 750	500	1.93 ^b^	*Synechococcus* sp. UTEX 2973	Cyanophyceae	[93]

* Data not available. ^a^ Approximate values obtained from figures. ^b^ Doubling time (h).

**Table 5:** Carotenoid accumulation at different light intensities.

Investigated Light Intensity, μmol photons m^−2^ s^−1^	Optimal Light Intensity for Carotenoid Synthesis, μmol photons m^−2^ s^−1^	Species and Strain	Carotenoid	Content in Dry Weight, mg g^−1^	Productivity, mg L^−1^ day^−1^	References
36.7, 69.5, 102.3	69.5	*Tetradesmus obliquus*	all carotenoids	374.30		[123]
21, 42, 63	63	*Nostoc calcicola*	all carotenoids	6.88		[124]
40, 160	40	*Phormidium* sp.	all carotenoids	4.62		[92]
1200		*Haematococcus lacustris* CCAP 34/7	Astaxanthin	590.31		[121]
350		*Haematococcus lacustris*	Astaxanthin	21.8		[125]
50, 100, 200, 400	400	*Chromochloris zofingiensis*	Astaxanthin	2.88	9.9	[122]
90, 460, 920	460, 920	*Chromochloris zofingiensis*	Astaxanthin	1.5		[126]
350		*Chromochloris zofingiensis*	Astaxanthin	6.3		[125]
350		*Coelastrella oocystiformis (=Scotiellopsis oocystiformis)*	Astaxanthin	6.4		[125]
350		*Neochloris wimmeri*	Astaxanthin	19.3		[125]
350		*Scenedesmus vacuolatus*	Astaxanthin	1.5		[125]
350		*Protosiphon botryoides*	Astaxanthin	14.3		[125]
1200		*Haematococcus lacustris* CCAP 34/7	Zeaxanthin	108.50		[121]
200, 500, 1000, 1500	1500	*Dunaliella salina* DF15	*all-trans* and *9-cis* β-carotene		3.5 and 2.3	[48]
200, 500, 1000, 1500	1500	*Dunaliella salina* DF17	*all-trans* and *9-cis* β-carotene		1.3 and 0.2	[48]
200, 500, 1000, 1500	1500	*Dunaliella salina* DF40	*all-trans* and *9-cis* β-carotene		2.6 and 2.0	[48]
200, 500, 1000, 1500	1500	*Dunaliella salina* CCAP 19/30	*all-trans* and *9-cis* β-carotene		3.2 and 0.2	[48]
200, 500, 1000, 1500	1500	*Dunaliella bardawil* UTEX 2538 (=*Dunaliella salina*)	*all-trans* β-carotene		2.9 and 2.2	[48]
200, 500, 1000, 1500	1000	*Dunaliella bardawil* UTEX 2538 (=*Dunaliella salina*)	*9-cis* β-carotene		2.9 and 2.2	[48]
1200		*Haematococcus lacustris* CCAP 34/7	β-carotene	59.99		[124]
33, 170, 280	33 (35 °C)	*Tetraselmis* sp. CTP4	β-carotene	4.41		[127]
33, 170, 280	170 (35 °C)	*Tetraselmis* sp. CTP4	β-carotene	3.21		[127]
33, 170, 280	280 (30 °C)	*Tetraselmis* sp. CTP4	Lutein	2.24		[127]
33, 170, 280	170 (35 °C)	*Tetraselmis* sp. CTP4	Lutein	3.17		[127]
90, 460, 920	90	*Chromochloris zofingiensis*	Lutein	4.0		[126]
92, 230, 368, 460, 690, 920, 1495	690	*Chlorella sorokiniana* *	Lutein	3.1		[128]
300		*Tetradesmus obliquus* FSP-3	Lutein		4.08	[78]

* 28 °C, 2 mM NaCl, 40 mM nitrate and under mixotrophic conditions.

**Table 6:** Effect of monochromatic LED illumination on microalgae.

LED	Light Intensity, μmol photons m^−2^ s^−1^	Species	Stimulating Effect in Decreasing Order					References
			Biomass	Lipid	Fatty Acids		Carotenoid	
					MUFA, PUFA	Individual Fatty Acids		
white, red, blue, green	60	*Botryococcus braunii*	red > white > blue > green	red > white > blue > green				[138]
white, red, blue, green, yellow	100	*Chlorella vulgaris*	white > red > blue > yellow > green			↑EPA, ↑DHA green > yellow		[139]
white, red, blue, green, yellow		*Chlorella vulgaris*	white > red > blue > yellow > green	blue > red > white > green > yellow			yellow > green > white > red > blue	[39]
white, red, blue, green, yellow		*Tetradesmus obliquus*	red > blue > white > yellow > green	blue > red > white > green > yellow			yellow > green > white > red > blue	[39]
red		*Tetraselmis suecica*				↑EPA		[24]
white, red, blue		*Chlorella vulgaris*, *Auxenochlorella pyrenoidosa* (=*Chlorella pyrenoidosa*), *Scenedesmus quadricaud*, *Tetradesmus obliquus*	blue > red > white		↑PUFA blue	↑ALA		[140]
white, red, blue, green, yellow	100	*Diacronema lutheri*	blue > red > green > yellow			↑EPA, ↑DHA yellow > green		[139]
white, red, blue, green, yellow	100	*Porphyridium purpureum* (=*Porphyridium cruentum*)	green > blue > yellow > red			↑EPA, ↑DHA red > blue		[139]
white, red, yellow		*Phaeodactylum tricornutum*	red > white > yellow	red > white		omega-3, omega-6 white > red		[72]
red		*Phaeodactylum tricornutum*			↓MUFA ↑PUFA			[72]
white, red, blue	52 ^a^	*Isochrysis galbana*	↑blue	↑blue		↑DHA blue		[141]
white, red, blue, green, yellow		*Arthospira platensis*	red > white > blue > yellow > green	blue > red > white > green > yellow			green > yellow > white > red > blue	[39]

Down arrow ↓ means decrease, up arrow ↑ means increase. ^a^ Intermittent light.

## Data Availability

Not applicable.
